# Comprehensive Assessment of Risk-Based Quality Management Adoption in Clinical Trials

**DOI:** 10.1007/s43441-024-00618-5

**Published:** 2024-02-16

**Authors:** Abigail Dirks, Maria Florez, Francois Torche, Steve Young, Brian Slizgi, Kenneth Getz

**Affiliations:** 1 Tufts Center for the Study of Drug Development, Tufts School of Medicine, Boston, MA USA; 2 CluePoints, Brussels, Belgium; 3 Price Waterhouse Coopers (PWC), London, United Kingdom

**Keywords:** Risk-based quality management, Risk-based monitoring, Quality by design

## Abstract

**Background:**

Risk-based monitoring (RBM) and risk-based quality management (RBQM) offer a compelling approach to increase efficiency, speed and quality in clinical trials by prioritizing and mitigating risks related to essential safety and efficacy data. Since 2013, the FDA and EMA have encouraged the use of RBM/RBQM, however adoption has been slow with limited understanding of the barriers to adoption.

**Methods:**

The Tufts Center for the Study of Drug Development conducted an online survey among pharmaceutical, biotechnology, and contract research organizations and gathered 206 responses on 32 distinct RBQM practices.

**Results:**

On average, companies implemented RBQM in 57% of their clinical trials. Lower levels of adoption were observed among companies conducting fewer than 25 trials annually (48%) compared to those conducting more than 100 trials annually (63%). Primary barriers to adoption include lack of organizational knowledge and awareness, mixed perceptions of the value proposition of RBQM, and poor change management planning and execution. Insights into improving the level of adoption are discussed.

## Introduction

Risk-based monitoring (RBM)—and its more expansive successor, risk-based quality management (RBQM)—offer a compelling approach to drive efficiency, speed and quality by prioritizing, preventing and mitigating clinical trial risks most associated with essential safety and efficacy data. For more than a decade, the European Medicines Agency (EMA) and the Food and Drug Administration (FDA) have encouraged the use of RBM and RBQM in the collection of clinical trial data via promotion of Quality by Design principles and the International Council for Harmonization (ICH) guidelines beginning with the second revision (R2) of E6 [1–3].

In June of 2023, the Food and Drug Administration (FDA) issued a draft guidance—and invited comment on the next iteration of ICH E6, which covers Good Clinical Practice. This third revision (R3) calls for even greater support of RBQM principles throughout clinical trial planning and execution. But despite more than a decade of encouragement from global regulatory agencies, there is only a limited understanding of RBQM adoption across the global drug development enterprise.

Recognizing the growing use of virtual and remote monitoring and data collection activity before and during the pandemic, the Association of Clinical Research Organizations (ACRO) conducted surveys among its member companies and found that 77% of clinical trials—ongoing at the end of 2020—had implemented at least one RBQM component, up from 47% of ongoing clinical trials in 2019 (before the pandemic) [4, 5].

The ACRO studies are a valuable initial assessment of RBQM adoption. The results were provided by six contract research organizations (CROs) drawn from their assessment of approximately 6000 clinical trials (6513 clinical trials in 2019; 5987 clinical trials in 2020). A total of eight primary RBQM components were evaluated: (1) Cross-functional risk assessment at the outset of the clinical trial; (2) Ongoing cross-functional risk assessment; (3) Use of quality tolerance limits (QTLs); (4) Use of key risk indicators (KRIs); (5) Use of centralized monitoring; (6) Use of off-site/remote-site monitoring; (7) Reduced source data verification (SDV); and (8) Reduced source data review (SDR). The ACRO study authors calculated the adoption rate of RBQM based on the percent of trials using only one of the eight components assessed [4–6].

The ACRO studies, however, have a number of limitations. CROs may be unaware of—and unable to report on—all RBQM activities implemented by sponsor companies, particularly those with substantial internal clinical, operating, and data management functions. Based on the EMA and FDA guidelines, there are arguably many more than eight primary RBQM activities that may be implemented during clinical trial planning and execution [1, 2]. A large heterogenous sample of sponsor organizations and a more granular set of RBQM components would provide for a measure of RBQM adoption that considers RBQM as a collection of multiple components and offers insight into varying levels of adoption by subgroup.

In the wake of the pandemic, there is a unique opportunity to build upon and update the ACRO assessment of RBQM adoption using a more comprehensive and complementary methodology based on direct report from pharmaceutical and biotechnology companies. This revised assessment can also gather new and up-to-date insight into barriers to adoption that tie to each direct report. Previous studies on the barriers to adoption were conducted pre-pandemic and are based on attitudinal surveys [7, 8].

To address this opportunity, in late 2022 through early 2023, the Tufts Center for the Study of Drug Development (Tufts CSDD) in collaboration with and funded by CluePoints and PwC conducted a more robust assessment of RBQM usage and barriers to adoption among pharmaceutical and biotechnology companies. Our methodology includes a comprehensive set of RBQM activities—32 distinct components in all—as well as a set of tools supporting these components.

## Methods

Tufts CSDD conducted an extensive review of the current literature and ICH E6 guidelines to design the data variables and collection process. A 29-question survey instrument comprised of close-ended and Likert-scale questions was developed including an assessment of 32 RBQM components and 8 tools organized around three primary sequential clinical trial stages: Planning and Design, Execution, Documentation and Resolution.

The stages were defined as follows: Planning & Design refers to the early stages of a clinical trial where research teams are designing the protocol, allocating resources, preparing a trial execution plan and engaging investigative sites. The Execution stage is comprised of ongoing activity after the clinical trial has been initiated. Documentation & Resolution includes identifying, evaluating, and reporting data, events, and risk observed during clinical trial execution.

Respondents were asked to estimate the percentage of trials within their organization’s portfolio that used each component. Respondents were also asked to estimate the percentage of clinical trials using artificial intelligence/machine learning to support RBQM. A list of the 32 RBQM components by each stage are included below. Questions about barriers to adoption, familiarity, trust, and commitment regarding RBQM were also presented. The survey was implemented between November 2022 and February 2023 using emails to a proprietary contact list of approximately 700 R&D executives in pharmaceutical and biopharmaceutical companies and contract research organizations globally.

### Components by Stage

#### Planning & Design

- Solicitation of input from patient community to support optimal trial design
- Solicitation of input from investigative research community to support optimal trial design
- Identification of critical-to-quality (CTQ) factors—to support optimal trial design
- Risk assessment—to support optimal trial design
- Identification of study-specific quality tolerance limits (QTLs)—during clinical trial design
- Assessment of protocol complexity—to support optimal trial design
- Identification of critical-to-quality (CTQ) factors—to support trial planning
- Identification of critical data and critical processes
- Risk assessment and risk control planning
- Identification of study-specific quality tolerance limits (QTLs)—during clinical trial planning
- Identification of study-specific key risk indicators (KRIs)
- Development of a centralized monitoring plan

#### Execution

- Key risk indicators (KRIs)
- Quality tolerance limits (QTLs)
- Statistical data monitoring
- Review of data visualizations
- Duplicate (aka “professional”) patients detection
- Reduced/targeted source data verification (SDV)
- Reduced/targeted source data review (SDR)
- Reduced/targeted on-site monitoring visits
- Remote site monitoring
- Reduced/targeted data management reviews
- Periodic review/update of risk assessment/risk control planning

#### Documentation & Resolution

- Identification of risks detected
- Evaluation, follow-up and resolution of each risk
- Identification of important QTL deviations
- Evaluation, follow-up and resolution of each important QTL deviation
- Updates made to definition of QTLs, KRIs, and other centralized monitoring risk detection components
- Updates to and/or deviations from centralized monitoring plan
- Data SDV’ed (source data verified)
- Data SDR’ed (source data reviewed)
- Updates to and/or deviations from site monitoring plan

Analyses of survey results were conducted to examine current adoption rates, gaps in adoption, differences by subgroup, and overall perspectives on the barriers to adoption and levels of trust and commitment to RBQM. Analyses were conducted by the Tufts CSDD research team using R (statistical software package). Current RBQM adoption rates were calculated as the mean aggregate percentage of clinical trials using RBQM components across organizations. Where respondents provided estimates for other components in the same section but left a particular component blank, the Tufts CSDD team assumed the organization was not using that component (i.e., that it was deliberately left blank). Analyses of variance (ANOVAs) were performed to compare the effect of annual trial volume, region, and company type on each of the 32 components, as well as aggregated percentages overall and by stage. Proportional differences in answers to perceptive questions were also tested for significant differences by subgroup.

## Results

A total of 206 respondents completed the survey representing a 30% response rate. Table 1 presents respondent characteristics. Over half of the respondents were from North America (58%), nearly a third were from Europe (31%), and the rest were from other countries, including Japan, China, Israel, India, Australia, Philippines, and South Africa (11%). More than half of the sample (55%) were clinical operations/clinical development professionals; 22% were data management and data science professionals; and the rest (23%) represented other functions. The majority (57%) were in Director/Manager level roles; C-Suite and senior vice president-level respondents were 8% and 16% of the total, respectively; and 18% were in other roles.

**Table 1:** Respondent characteristics

	Percent of total (*n*)
World Region	
North America	58% (88)
Europe	31% (47)
ROW (includes Japan, China, Israel, India, Australia, Philippines, South Africa)	11% (17)
Company Type	
Pharma/biotech	78% (121)
CRO	15% (23)
Med device/diagnostics and vendors	7% (11)
Annual Trial Volume	
Less than 25	33% (50)
25 to 100	36% (54)
More than 100	31% (46)
Functional Area	
Clinical operations/clinical development	55% (85)
Data management/data science	22% (34)
Other	23% (36)
Role within Organization	
C-Suite	8% (12)
SVP	16% (25)
Director/manager	57% (88)
Other	18% (28)

Respondents represented an estimated 125 distinct companies (differentiated with proxy measures from survey responses), 78% were pharmaceutical or biotechnology companies, 15% were contract research organizations (CROs), and 7% were medical device or vendors. In all, respondents provided estimated adoption levels for more than 12,000 total clinical trials. Companies were evenly distributed by trial volume per year: approximately one-third (33%) conducted less than 25 clinical trials annually; another one-third (36%) conducted 25 to 100 each year; and the remaining one-third (31%) conducted more than 100 trials per year (Table 1).

### RBQM Adoption

Tables 2 and 3 present adoption levels by subgroup overall and by stage. The overall mean adoption level across all companies in the sample, including all RBQM components, was 57%. The adoption of RBQM components in 2023 is highest in the Documentation stage, at 60%, followed by Planning & Design at 56%. Components in the Execution stage had the lowest reported level of adoption at 52% of all ongoing clinical trials. Companies conducting less than 25 trials per year had an overall lower RBQM adoption rate of 48% when compared to companies conducting more than 25 clinical trials annually at 59% (25–100 clinical trials) and 63% (more than 100 clinical trials), respectively (*p* < 0.001). European companies had significantly higher adoption rates at 64% than companies in the Rest of the World at 45% (*p* = 0.04). Although not statistically significant, European companies also had higher reported adoption rates in all stages compared to North America.

**Table 2:** Overall RBQM Adoption Rates by Each Subgroup

	Components				Tools		
	Planning & design (%)	Execution (%)	Documentation & resolution (%)	All components in aggregate (%)	Planning & design (%)	Execution (%)	RBQM tools (%)
Overall	56%	52%	60%	57%	48%	34%	46%
Annual Trial Volume							
< 25^a^	47%	41%	57%	48%	34%	23%	31%
25 to 100	62%	54%	62%	59%	47%	32%	43%
100 +	59%	63%	62%	63%	52%	47%	50%
Region							
Europe	64%	59%	69%	64%	48%	37%	43%
North America	53%	50%	57%	54%	45%	33%	41%
ROW	42%	43%	52%	45%	32%	31%	32%

^a^RBQM adoption is lowest in companies with < 25 trials per year (Components: *p* < 0.001, Tools: *p* < 0.01)

**Table 3:** Individual Component Use Across Clinical Trials by Stage

Components	Actual^a^ (2023) percent of clinical trials	Expected (2027) percent of clinical trials	Growth/decline in usage (2023–2027) (%)
Planning & Design			
Solicitation of input from patient community to support optimal trial design	27	63	133%
Solicitation of input from Investigative Research community to support optimal trial design	57	80	40%
Identification of critical-to-quality (CTQ) factors—for clinical trial design	55	87	58%
Risk assessment to support optimal trial design	65	86	32%
Identification of study-specific quality tolerance limits (QTLs)—for clinical trial design	49	80	63%
Assessment of protocol complexity to support optimal trial design	51	80	57%
Identification of critical-to-quality (CTQ) factors—for clinical trial planning	47	85	81%
Identification of critical data and critical processes	68	91	34%
Risk assessment and risk control planning	79	94	19%
Identification of study-specific quality tolerance limits (QTLs)—for clinical trial planning	48	82	71%
Identification of study-specific key risk indicators (KRIs)	62	88	42%
Development of a centralized monitoring plan	46	86	87%
Execution			
Key risk indicators (KRIs)	63	88	40%
Quality tolerance limits (QTLs)	46	82	78%
Statistical data monitoring	49	78	59%
Review of data visualization	59	85	44%
Duplicate (aka “professional”) patients detection	24	56	133%
Reduced/targeted SDV	56	84	50%
Reduced/targeted SDR	46	76	65%
Reduced/targeted on site monitoring	54	83	54%
Remote site monitoring	60	82	37%
Reduced/targeted data management reviews	38	73	92%
Periodic review/update of risk assessment/risk control planning	64	91	42%
Documentation & Resolution			
Identification of risks detected	76	94	24%
Evaluation, follow-up and resolution of each risk	70	91	30%
Identification of important QTL deviations	46	82	78%
Evaluation, follow-up and resolution of each important QTL deviation	44	80	82%
Updates made to definition of QTLs, KRIs, and other centralized monitoring risk detection components	41	77	88%
Updates to and/or deviations from centralized monitoring plan	48	77	60%
Data SDV’ed (source data verified)	76	66	− 13%
Data SDR’ed (source data reviewed)	72	69	− 4%
Updates to and/or deviations from site monitoring plan	65	78	20%

Overall tool usage was at 46% of trials across organizations. Similar to component usage, RBQM tools were implemented in a lower proportion of trials, on average, in companies conducting fewer than 25 trials per year when compared to those conducting 25 to 100 trials or 100 or more trials per year (*p* < 0.01).

In the Planning & Design stage the most widely used individual components were risk assessment and risk control planning (79%) and identification of critical data and critical processes (68%). The component used in the lowest proportion of trials, on average, was solicitation of input from patient community to support optimal trial design at 27%, but expected to increase the most, with an expected percent change of 133%.

Use of individual RBQM components in the Execution stage range from 24% (for duplicate (aka “professional”) patients detection)  to 64% (for periodic review/update of risk assessment/risk control planning) of all clinical trials. Between 46% and 56% of clinical trials are using reduced/targeted source data review and verification. Companies project that use of all RBQM components in the Execution stage will increase by 2027 with the highest expected growth in the use of duplicate (aka "professional") patients detection and reduced/targeted data management reviews.

Within the Documentation and Resolution stage, documentation of data SDV’ed (source data verified) and identification of risks detected is being conducted in 76% of clinical trials on average. The documentation of data SDV'ed (source data verified) is expected to decrease by 13%. Similarly, data SDR'ed (source data reviewed)  is documented in 72% of trials but expected to decrease by 4% by 2027. The least used component was updates made to definitions of QTLs, KRIs, and other centralized monitoring risk detection components, at 41% of trials, on average.

### Respondent Perspectives

Table 4 presents respondent perceptions and attitudes overall and by world region, company type, annual trial volume, and functional area. Nearly all (96%) of respondents indicated that they are ‘somewhat’ or ‘very’ familiar with RBQM. The majority of respondents (83%) indicated that their organization is ‘somewhat’ or ‘very’ committed to supporting RBQM. Approximately three-out-of-four respondents (76%) reported having understanding of the term RBQM.

**Table 4:** Reported Familiarity, Understanding and Perceived Value proposition Overall and by Subgroup

Percent Indicating ‘Somewhat’ or ‘Very’	Familiarity with the term RBQM (%)	Understanding of the term RBQM ^a^ (%)	Trust in improving quality of clinical research (%)	Trust in increasing efficiency and cost savings (%)	Trusting in reducing study timelines (%)	Commitment to RBQM (%)
Overall	96%	76%	78%	63%	53%	83%
World Region						
North America	94%	70%	75%	56%	49%	80%
Europe	98%	85%	74%	65%	50%	85%
ROW	100%	82%	94%	88%	65%	82%
Company Type						
Pharma/biotech	96%	74%	75%	61%	46%	79%
CRO	100%	78%	82%	57%	74%	96%
Med device/diagnostics and vendors	100%	88%	100%	89%	78%	100%
Annual Trial Volume						
< 25	94%	59%	69%	43%	37%	71%
25 to 100	96%	86%	84%	69%	57%	90%
> 100	100%	81%	79%	73%	62%	85%
Functional Area						
Clinical operations/clinical development	98%	73%	81%	61%	57%	84%
Data management/ data science	97%	85%	73%	70%	42%	82%
Other	94%	72%	74%	57%	50%	80%

^a^How well would you say your organization understands what RBQM means?

With regards to the perceived value proposition of RBQM, nearly eight-out-of-ten (78%) trust that RBQM will improve the overall quality of research; 63% trust that RBQM will enable efficiency and cost savings; and 53% trust that RBQM will reduce clinical trial timelines.

Respondents selected the top-three barriers to RBQM adoption within each stage of the implementation process. The top reported barrier is ‘lack of knowledge’ of RBQM, followed by ‘investment of time’ and ‘lack of skills’. Aggregating across all stages, every subgroup analyzed mentioned ‘lack of knowledge’ as a top-three barrier, and more often than other barriers. Respondents in Europe noted ‘investment of time' most often (55%) after ‘lack of knowledge.’ Those in North America mentioned ‘lack of skills’ most often (50%) after ‘lack of knowledge’. Cost was a top-three barrier for approximately half of pharma/biotech respondents (51%) while CROs mentioned ‘lack of skills’ (54%) and ‘investment of time’ (43%) more often than they mentioned cost (40%). When looking at barriers by annual trial volume, ‘lack of knowledge’ emerged again as a top barrier in all trial volume categories. More than half of companies conducting fewer than 25 trials per year noted ‘investment of time’ as a barrier, while more than half of companies conducting 25 to 100 trials per year noted ‘lack of skills’ as a barrier. More than half of companies conducting 100 trials or more per year noted ‘lack of technology’ as a barrier. Differences in perceptions about barriers were also found by functional area. Those in clinical operations / clinical development and data management/data science reported ‘lack of knowledge’, ‘investment of time’, and ‘lack of skills’ as top barriers to implementation. See Table 5.

**Table 5:** Top Barriers to Adoption Reported by Subgroup—percentage of respondents selecting each barrier

	Lack of knowledge (%)	Investment of time (%)	Lack of skills (%)	Lack of technology (%)	Lack of organizational consensus (%)	Cost (%)
Overall	69%	48%	47%	42%	38%	26%
World Region						
North America	68%	46%	50%	46%	39%	28%
Europe	69%	55%	46%	37%	36%	17%
ROW	58%	27%	51%	45%	54%	27%
Company Type						
Pharma/biotech	67%	49%	47%	45%	38%	51%
CRO	66%	43%	54%	34%	43%	40%
Med device/diagnostics and vendors	74%	26%	55%	47%	21%	0%
Annual Trial Volume						
< 25	57%	52%	41%	47%	36%	38%
25 to 100	72%	45%	58%	34%	39%	24%
> 100	73%	46%	46%	52%	40%	11%
Functional Area						
Clinical operations/clinical development	68%	49%	51%	44%	37%	23%
Data management/data science	68%	45%	42%	34%	39%	26%
Other	66%	42%	48%	47%	39%	28%

Table 6 presents the functional areas considered to be the most reluctant to adopt and embrace RBQM. Respondents report that site management and investigative site monitoring are the most reluctant followed by clinical development and clinical operations/study management functions. Biostatistics was mentioned the least, at 22% of respondents. The majority of respondents (75%) noted that more experience with successful implementations would increase trust and commitment, and a little less than half said that communication (45%) and more training (44%) would help increase trust. Only 17% said that better incentives would help increase trust. Table 7 presents the full list of choices that respondents indicated would help increase trust in RBQM.

**Table 6:** Respondent Perspectives—Functional Areas with the Least Trust in RBQM

In what functional areas is trust in RBQM low?	% (*n*)
Site management/site monitoring	43% (62)
Clinical development	43% (61)
Clinical operations/study management	42% (60)
Executive leadership	37% (53)
Data management/data sciences	27% (39)
Biostatistics	22% (32)

**Table 7:** Respondent Perspectives—How to Increase Trust in RBQM

What would help increase trust in RBQM in your organization?	% (*n*)
More experience with successful implementations	75% (116)
Communication	45% (69)
More training	44% (68)
Mandated use within organization	37% (57)
More elaborate change management plan	33% (51)
High quality vendor	26% (40)
Better incentives	17% (26)

## Discussion

The results of this more comprehensive and robust assessment indicate that overall, sponsor and CRO companies are implementing RBQM components on slightly more than half (57%) of their clinical trials, on average, in 2023. The highest levels of reported adoption are in the Documentation and Resolution stage (60% of clinical trials) in contrast with the lowest reported levels of adoption in the Execution Stage (52% of clinical trials). The overall and individual stage levels of adoption are well below the 77% level reported during the pandemic in the ACRO study.

Strong organizational commitment tempered by mixed levels of awareness and perceptions about RBQM’s ability to deliver higher efficiency, lower cost and faster speed help explain the current reported levels of adoption. The interpretation of some results might be impacted by respondent’s misunderstanding of what RBQM really means. An industry led initiative to get to a common set of terms together with consistent definitions would contribute to more accurate assessments of usage levels and barriers to adoption.

Barriers and resistance to adoption are most prominent in clinical development and clinical operations—including site management—functions that are especially focused on clinical trial efficiency outcomes and cost savings. Some have suggested that these functions may also be the most threatened by RBQM’s potential to reduce and facilitate a more targeted role for site monitoring.

Significant variation in adoption is observed by company size (e.g., annual clinical trial volume) with the larger organizations reporting higher levels of adoption overall and by individual RBQM components. Sponsor companies with larger R&D portfolios are dedicating more infrastructure and standardized operating and reporting procedures to their data and risk management practices. Although not statistically significant, the higher relative adoption of RBQM components in Europe compared to North America and the Rest of World, across all three stages, is also notable and may reflect the European community’s particular sensitivity and commitment to risk planning, assessment, and reporting. The study findings offer important new benchmarks on the use of 32 individual RBQM components by several major subgroups that can be tracked over time.

Perceptions on the primary barriers to adoption including lack of knowledge and awareness, lack of organizational consensus and the lack of skills all point to the need for more effective change management practices. Indeed, respondents specifically noted the importance of communicating successful RBQM implementations, better training and more elaborate change management planning as opportunities that would help increase organizational trust in and use of RBQM components.

The study findings suggest that communication and education should be tailored to functional areas. Clinical and clinical operations functions, in particular, have lower relative reported levels of understanding and lower confidence in the ability of RBQM to deliver cost and efficiency improvement.

It is important to note that although assessment of RBQM adoption in this study is more robust, it is still based on respondent opinion and estimation. As such, within especially large organizations, respondents may have had limited line of sight into all components being utilized across functions. Respondent self-report is also based on retrospective activity. Future research might gather prospective data on specific clinical trials to inform understanding of current and anticipated levels of RBQM adoption. Tufts CSDD intends to publish additional findings from this new study in subsequent manuscripts including a more detailed look at specific tools supporting RBQM adoption and the current and expected role of AI-enabled approaches.

## Conclusion

The results of this study indicate that risk-based quality management components are used on approximately half of all clinical trials with the most common reported areas of use observed in the documentation and resolution of risk, followed by planning and design areas including the identification of risk factors, risk assessment to support optimal design and risk control planning. The adoption levels found in this study are below those of other recent studies. Company size, and to a lesser extent geographic location, are associated with higher observed levels of adoption. Although most organizations are committed to supporting RBQM, current levels of adoption are explained in part by mixed views on the value proposition of RBQM—specifically its ability to deliver efficiency, lower cost and faster clinical trials—and limited and insufficient change management practices. The results of this study suggest that there are opportunities to drive adoption including developing consensus definitions and terms, publicizing and sharing explicit case examples characterizing successful RBQM experiences and offering targeted training to improve awareness and understanding of the value of RBQM.

## Data Availability

It is Tufts CSDD’s policy to make de-identified raw data available for scholarly analysis.
