# Correction: Comprehensive Assessment of Risk-Based Quality Management Adoption in Clinical Trials

**DOI:** 10.1007/s43441-024-00648-z

**Published:** 2024-04-30

**Authors:** Abigail Dirks, Maria Florez, Francois Torche, Steve Young, Brian Slizgi, Kenneth Getz

**Affiliations:** 1 Tufts Center for the Study of Drug Development, Tufts School of Medicine, Boston, MA USA; 2 CluePoints, Brussels, Belgium; 3 Price Waterhouse Coopers (PWC), London, UK

**Correction: Therapeutic Innovation & Regulatory Science**

10.1007/s43441-024-00618-5

The article Comprehensive Assessment of Risk-Based Quality Management Adoption in Clinical Trials, written by Abigail Dirks, Maria Florez, Francois Torche, Steve Young, Brian Slizgi, and Kenneth Getz, was originally published electronically on the publisher’s internet portal on February 16 2024 without open access. With the author(s)’ decision to opt for Open Choice the copyright of the article changed on March 20 2024 to © The Author(s) 2024 and the article is forthwith distributed under a Creative Commons Attribution CC BY.

